# Anti-hyperglycemic, anti-hyperlipidemic, and anti-inflammatory effect of the drug Guggulutiktaka ghrita on high-fat diet-induced obese rats

**DOI:** 10.1016/j.jaim.2022.100583

**Published:** 2022-06-24

**Authors:** Samreen M. Sheik, Pugazhandhi Bakthavatchalam, Revathi P. Shenoy, Basavaraj S. Hadapad, Deepak Nayak M, Monalisa Biswas, Varashree Bolar Suryakanth

**Affiliations:** a Department of Biochemistry, Kasturba Medical College, Manipal, Manipal Academy of Higher Education, Manipal, Karnataka, 576104, India; b Department of Anatomy, Melaka Manipal Medical College, Manipal, Manipal Academy of Higher Education, Manipal, Karnataka, 576104, India; c Division of Ayurveda, Centre for Integrative Medicine and Research, Manipal Academy of Higher Education, Manipal, Karnataka, 576104, India; d Department of Pathology, Kasturba Medical College, Manipal Academy of Higher Education, Manipal, Karnataka, 576104, India

**Keywords:** Guggulutikthaka gritha, High fat diet, Hyperglycemia, Hyperlipidemia, Inflammation, ASCVD, Atherosclerotic cardiovascular disease, CVD, Cardiovascular disorders, CRP, C-reactive protein, DM, diabetes mellitus, GTG, Guggulutikthaka gritha, HDL, High-Density Lipoprotein, HFD, High fat diet, ICAM-1, Intracellular adhesion molecule 1, IL-1, IL- 6, IL-10, Interleukins, IR, Insulin resistance, LDL, Low-density lipoprotein, LPL, Lipoprotein lipase, OECD, Organization for economic co-operative and development, oxLDL, Oxidized LDL, ROS, Reactive Oxygen Species, TG, Triglycerides, TLR, Toll-like receptors, TNF-α, Tumor necrosis factor, VCAM 1, Vascular cell adhesion molecule – 1, VLDL, Very-low-density lipoprotein

## Abstract

**Background:**

Ayurveda is a holistic system of medicine and describes a vast array of herbs and herbal mixtures that are been demonstrated to possess efficacy in research investigations. Guggulutikthaka gritha (GTG) is one such drug evaluated for its role in skin and bone diseases.

**Objective:**

In the current study, the hypoglycemic, hypolipidemic, and anti-inflammatory effect of the drug GTG was studied with the scope to treat dyslipidemia and thereby reduce the risk of cardiovascular disease.

**Materials and method:**

The animals (Wistar rats) were fed a high-fat diet and dyslipidemia was induced. The control group was provided with a normal chow diet and had free access to water. The treatment with the drug GTG was given for 21 days after confirming dyslipidemia. The blood glucose was measured immediately using a glucometer. The serum was analyzed for lipid profile and Vascular Cell Adhesion Molecule – 1(VCAM 1) by ELISA method before and after treatment. The histopathology of the heart and liver was also performed.

**Results:**

The abnormal change in lipid profile, blood glucose, and inflammatory marker along with the accumulation of intracellular fats in the arteries of the heart and liver confirmed dyslipidemia. A significant reduction in serum lipid profile (p < 0.05), blood glucose (p < 0.05), and VCAM 1 (p < 0.05) was noted after the treatment with significant histopathological changes in arteries of the heart and liver.

**Conclusion:**

The study provides scientific validation on the drug GTG being effective in hyperglycemia, hyperlipidemia, and inflammation in dyslipidemia.

## 1 Introduction

An increase in body weight due to excessive food intake and low energy output leads to obesity [1]. During obesity macrophages infiltrate into the fat, bringing about a shift in their phenotype from anti-inflammatory M2 to pro-inflammatory M1 [2]. The changes in fat composition lead to the development of adiposopathy (adipose tissue dysfunction) and altered adipokines secretion which is liable to cause obesity-related metabolic disorders [1].

There is a strong relationship between dyslipidemia and type 2 diabetes, obesity, and heart disease [3]. Dyslipidemia that occurs in obese patients is characterized by increased TG and free fatty acids, decreased HDL-C, normal or slightly elevated LDL-C with more proatherogenic composition (small dense LDL) [4].

Accretion of lipids mainly LDL leads to inflammation which stimulates the endothelium to release trans-membrane proteins such as TLR and T cells. The TLR initiates adhesion molecules such as VACM-1, ICAM-1, and P-selectin beside the release of macrophages. The macrophages with the assistance of myeloperoxidase release inflammatory cytokines (TNF-α, IL-1, IL-6, IL-10, and CRP). The cytokines excite ROS that converts the LDL to oxLDL, secreting Monocyte-Colony Stimulating factor, which gets modified as foam cells that block the artery leading to atherosclerosis [5,6].

Obesity remains the most significant unfavorable consequence despite preventive and therapeutic efforts. The treatment of obesity co-morbidities must be a central objective of care to reduce the risk for cardiovascular and other chronic complications [7].

The statin inhibits 3-hydroxy-3-methylglutaryl coenzyme-A reductase, competently blocking liver cholesterol synthesis and lowering LDL-C by as much as 50% from baseline consistent with statin effectiveness [8]. Over the past several decades, statins have been the standard of care for dyslipidemia. ASCVD risk is reduced by 15%–37% by statins, but the residual risk of 60%–80% remains [9]. Despite optimal statin therapy, these remaining ASCVD risk factors have been found to cause major vascular events in 20% of patients with coronary heart disease [10].

Hence the recognition of complementary medicine has vividly increased in many countries to control dyslipidemia. This might be accredited to the aging of the population, the circumstance of habitual conditions, and agony about the adversative effect of chemical drugs [11].

The system of Ayurvedic medicine originated on the Indian subcontinent 3000 years ago [12]. Ayurvedic lipid formulation, GTG also called Panchatikta Guggulu ghrita and Nimbadi ghrita in the classical ancient text Astanga hridayam, has been used in the treatment of bone diseases [13] and has been included in the Ayurvedic pharmacopeia of India (A.F.I. Part-I, 6:27) [14] as a remedy for chronic inflammatory conditions such as Sandhigata Vata, Vatarakta, Asthigata Vata (Osteoarthritis, Gout, Osteoporosis respectively). Due to the lipid base, ghritas are traditional Ayurvedic formulations prepared in ghee with polyherbal decoctions, which allows better absorption and delivery of the active constituents. In ayurvedic medicine, since most polyherbal herb has many different medicinal properties, they will affect multiple metabolic pathways [15]. Research on lipid-lowering and anti-inflammatory effects are relatively limited.

The present study aimed to assess the hypoglycemic, hypolipidemic, and anti-inflammatory effects of the drug Guggulutiktaka ghrita with the scope to treat dyslipidemia and thereby reduce cardiovascular disease.

## 2 Materials and methods

### 2.1 Animals

An Institutional Animal Care and Use Committee reviewed and approved the study protocol (IAEC/KMC/111/2018). Study subjects included 12 male Wistar rats weighing 150–200 g kept in a 12 h light/12 h dark cycle, fed wheat gluten feed pellets, and provided with water.

### 2.2 Drug material

The drug GTG was prepared at Shree Dharmasthala Manjunatheshwara Ayurveda Pharmacy, Udupi, Karnataka, India. The testing laboratory for ayurvedic formulation preparation follows the AYUSH guidelines and is a GMP-certified laboratory (License no. AUS 783 dated 25th September 2015). The drug was prepared according to the classical method of preparation using the following ingredients: *NimbaTwak*, *Guduchi*, *vasaka*, *Patola*, *Kantakari*, *Patha*, *Vidhanga*, *Devadaru*, *Gajapippali*, *Yavakshara*, *Sarjakshara*, *Nagara*, *Kushta*, *Haridra*, *Shatapushpa*, *Chavya*, *Tejovati*, *Maricha*, *Kutaja*, *Yavani*, *Chitraka*, *Katuki*, *ShudhaBallataka*, *Vacha*, *PippaliMoola*, *Yukta-Rasna*, *Ativisha*, *manjishta*, *Vishani-Rishabha*, *Guggulu*, *water* (as it is), *Ghritha* (as it is) [16].

### 2.3 Acute toxicity/lethal dosage study of the drug GTG

It was done according to the OECD guidelines 425 [17]. The animals were fasted overnight (deprived of food but not water).

• At first, the limit test was performed, and an animal was administered with 1 ml/100 g body weight of the drug and observed continuously for 30 min, 1 h, and then frequently for 4 h, 8 h, 12 h, 24 h, and 48 h. The animal survived and with an interval of 48 h, another animal was dosed, continued for 5 animals, and then observed for 14 days.
• Irwin's test was performed where the animals were witnessed for gross behavioral alterations in the parameter such as Behavioral profile, Neurological profile, and autonomic profile.
• 1/10th of the maximum accepted harmless dose of the drug from the acute toxicity study was selected for further study.

### 2.4 Induction of dyslipidemia

A high-fat diet was given to male Wistar rats (6–8 weeks old) for 11 months. The diet contained 33.8% fat, 27.1% carbohydrates, and 23.9% protein, along with 5.8% fiber, 6.2% moisture, and 3.2% vitamins and minerals [18,19]. Food and water were accessed freely by the rats. After 11 months of stabilization with HFD, confirmation of dyslipidemia was done. The rats were withdrawn from the food for about 8 h and the blood was withdrawn intraorbitally using mucap capillary. About 1.5 ml of blood was collected in a red vacutainer and the serum was separated after centrifugation for biochemical analysis. Along with this, a rat was sacrificed in each group and histological study of the heart arteries and liver was done to check for lipid deposition and inflammation.

### 2.5 Experimental design and treatment schedule

After confirmation of dyslipidemia, 0.1 ml of the drug was given to the test group for 21days [20]. On the 22nd Day, the blood was withdrawn after overnight fasting of 12 h (deprived of food but not water). Serum was used for the analysis of biochemical and immunological parameters. A rat in the group was sacrificed and the artery of the heart and liver was isolated for histology. Bodyweight was monitored weekly.

### 2.6 Biochemical analysis

The serum was used to analyze Total cholesterol by Cholesterol oxidase peroxidase method, TG by glycerol phosphate oxidase/peroxidase enzymatic colorimetric method, and HDL-C by direct enzymatic method. The Coral Clinical Systems kits were used, and the assay was performed using Erba Semi-Auto Biochemistry analyzer. The blood glucose was analyzed using a standard glucodot glucometer device during the collection of blood from intraorbital puncture.

### 2.7 Analysis of immunological marker

The serum was analyzed for Rat VCAM-1 by Enzyme-linked immunosorbent assay using Robonic ELISA plate reader analyzer.

### 2.8 Histology

A paraplast block was prepared from serial 5 mm slices of the common carotid artery for analysis of fat accumulation in the heart after its fixation in neutral buffered formalin for 8 h. Light microscopy was used to visualize every 5-micron section of the tissue block stained with Hematoxylin and Eosin (H&E) stain. Similarly, three sections from the liver were analyzed for histomorphologic changes predominantly to confirm light microscopic manifestations of dyslipidemia [21].

### 2.9 Statistical analysis

Statistical analyses were conducted using SPSS 16.0 software (SPSS Inc., India) and results were expressed in mean ± SD for n animals. Comparing the two groups was done using the Paired t-test. Statistical significance was determined at P < 0.05.

## 3 Results

### 3.1 Standardization of the drug GTG

The pure medicated ghee obtained after the classical method of preparation is used after the standardization/quality control checks performed for the components used and the final preparation of the test material (Supplementary data).

### 3.2 Acute toxicity/lethal dosage study of the drug GTG

The drug GTG was found to be non-toxic and 0.1 ml of the safe dose was selected for the study as per the limit test conducted according to OECD guidelines 425 and no behavioral changes were recorded as per the Irwin's test.

### 3.3 Induction of dyslipidemia

After stabilizing the dyslipidemic group with HFD for 11 mon, the serum TC, TG, VLDL was significantly increased (p < 0.05) along with increase in HDL (p < 0.005) was noted. The increase in blood glucose levels (p < 0.05) and the inflammatory cell adhesion molecule, VCAM-1(P < 0.005) is also seen. The above result indicated hyperlipidemia, hyperglycemia and inflammation leading to the development of dyslipidemia (Table 1).

**Table 1:** Comparison of serum lipids, blood glucose, and serum VCAM-1 level between the two groups.

Test parameter	NC	DC
TC (mg/dL)	80.83 ± 20.92	156.33 ± 39.42#
TG (mg/dL)	116 ± 46.76	202 ± 81.61∗
HDL (mg/dL)	25.8 ± 10.83	94.16 ± 22.78^#^
LDL (mg/dL)	31.83 ± 17.40	42.73 ± 27.59
VLDL (mg/dL)	23.20 ± 9.35	40.43 ± 16.32∗
Blood Glucose (mg/dL)	103.33 ± 8.93	137 ± 20.73∗
VCAM 1 (ng/mL)	5.52 ± 1.26	8.25 ± 1.02^#^

NC = Normal Control, DC = Dyslipidemic control.

∗ = P < 0.05, # = P < 0.005 compared to normal controls.

Values are expressed in mean ± SD.

### 3.4 Treatment

After 21 days of treatment with GTG, the dyslipidemic group significantly reduced their TC (p < 0.005), TG (p < 0.05), VLDL (p < 0.05), blood glucose (p < 0.005), and inflammatory marker VCAM-1 (p < 0.005) indicating that GTG has an anti-hyperlipidemic, anti-hyperglycemic, and anti-inflammatory effect on them.

### 3.5 Histology

**Fig. 1:**
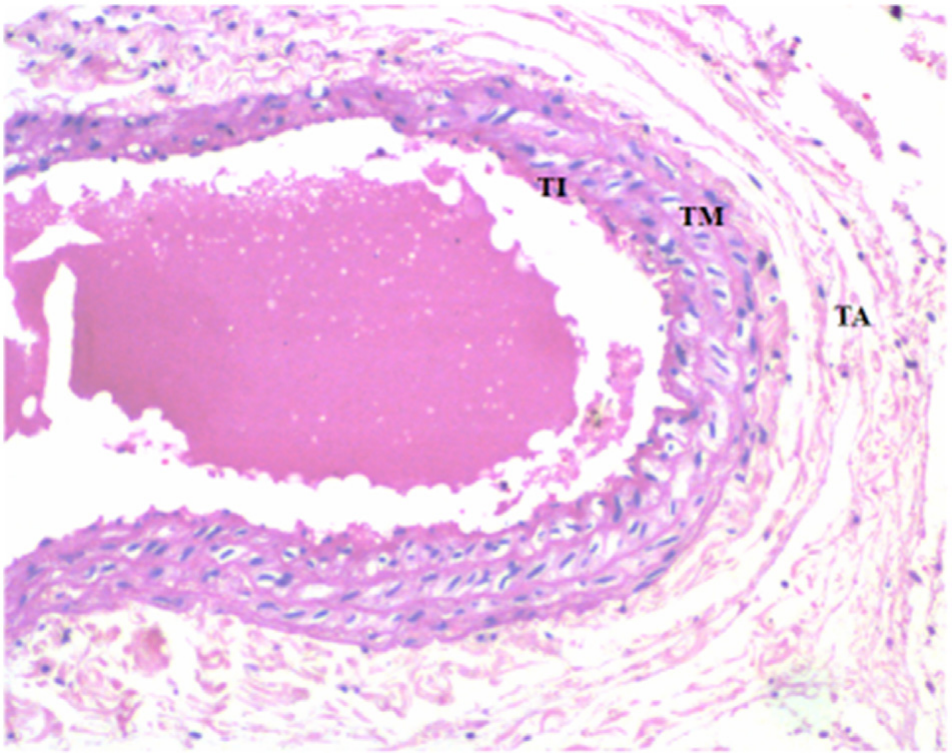
Microscopic image of Hematoxylin and Eosin stained Normal arterial wall (10X), TI- Tunica Intima, TM- Tunica Media, TA- Tunica Adventitia.

**Fig. 2:**
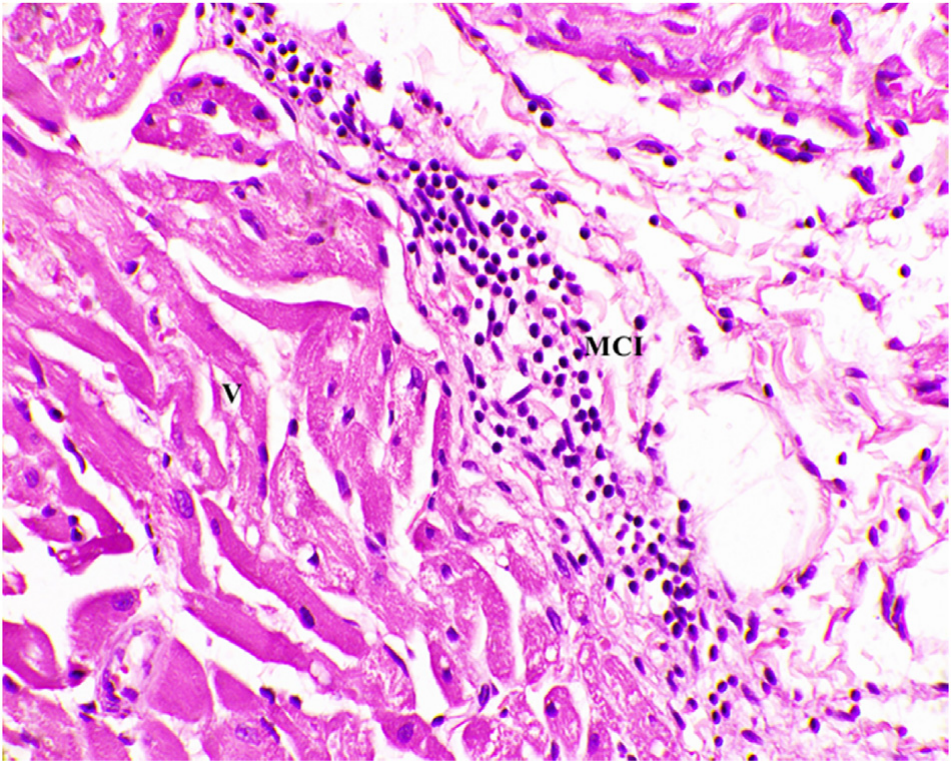
Microscopic image of Hematoxylin and Eosin stained diseased arterial wall showing mononuclear cell infiltration, arterial vacuoles hyalinosis, and luminal narrowing (40x). V- Vacuoles, MCI- Mononuclear Cell Infiltration.

The microscopic images of hematoxylin and eosin stained arterial tissues showed fatty degeneration in the arterial wall. Micro and macro vascular degeneration with intracellular fat droplets were also noted in the arteries of the heart (Fig. 2) in comparison to the normal control (Fig. 1). This finding is suggestive of development of dyslipidemia in the vessels of the heart.

**Fig. 3:**
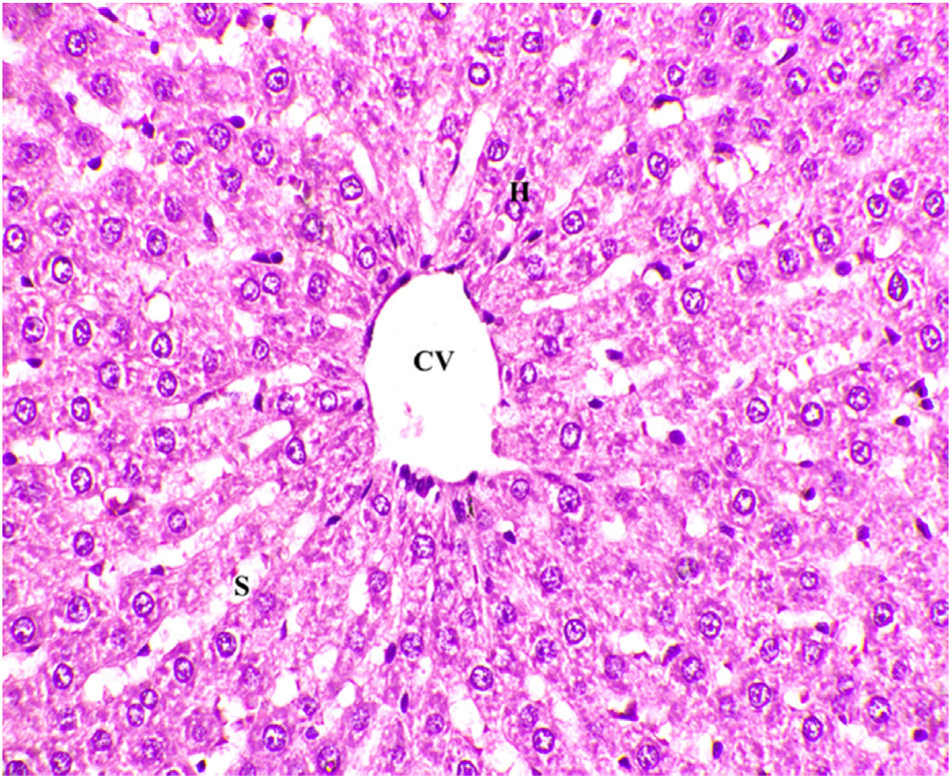
Microscopic image of Hematoxylin and Eosin stained normal liver tissue (40x). CV- Central Vein, H- Hepatocytes, S- Sinusoids.

**Fig. 4:**
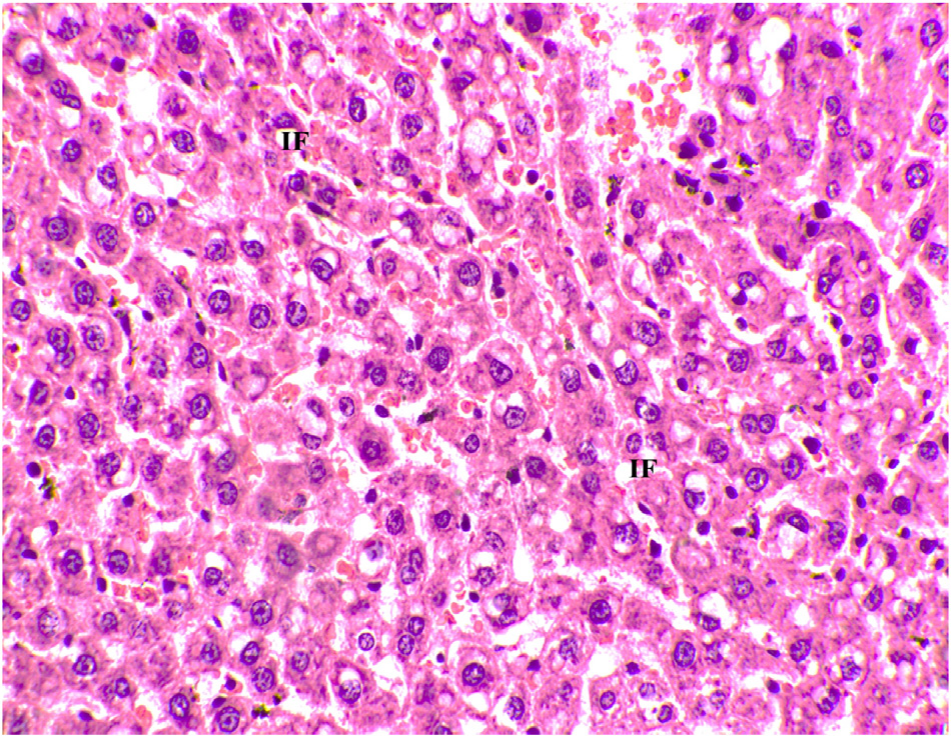
Microscopic image of Hematoxylin and Eosin stained diseased liver tissue showing intracellular fat deposits (40x). IF- Intracellular Fat.

The microscopic images of hematoxylin and eosin stained liver tissues showed excess accumulation of fat cells (Fig. 4) in comparison with the normal control (Fig. 3). The above histopathological results of the liver tissue confirms the development of dyslipidemia in the liver.

**Fig. 5:**
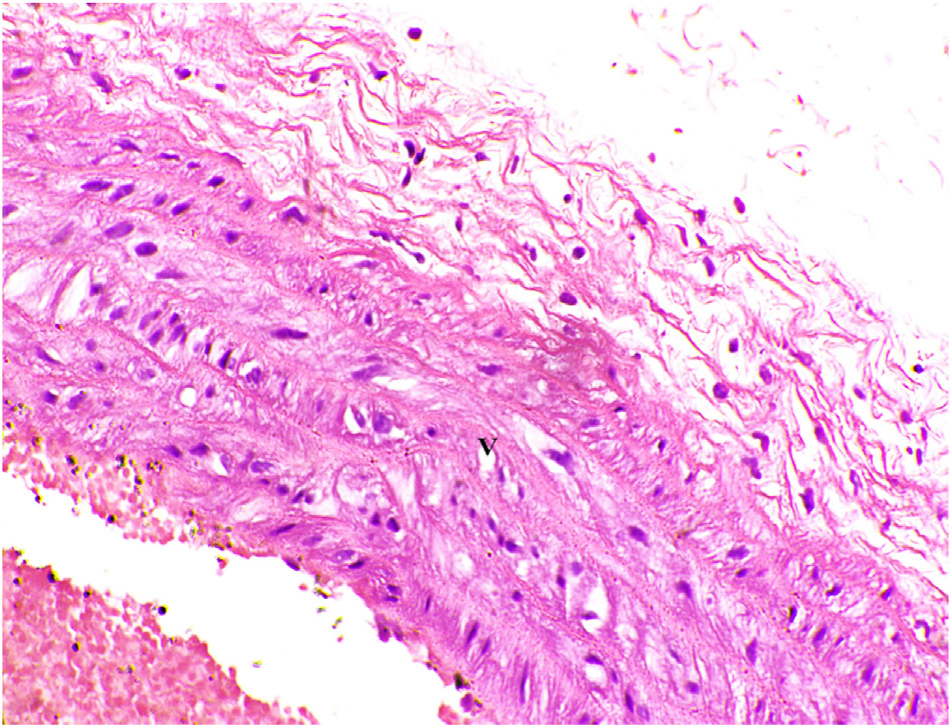
Microscopic image of Hematoxylin and Eosin stained arterial wall showing reduced mononuclear cell infiltration, arterial vacuoles after treatment (40x). V- Vacuoles.

**Fig. 6:**
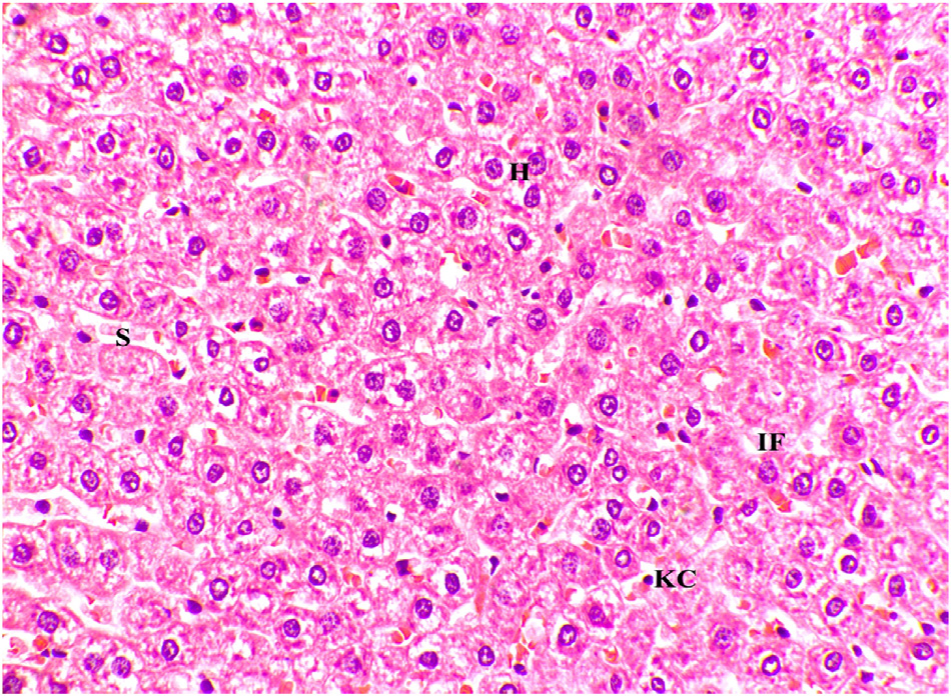
Microscopic image of Hematoxylin and Eosin stained liver tissue after treatment with GTG (40x). H- Hepatocytes, IF- Intracellular Fat, S – Sinusoids, KC – Kupffer Cells.

The histology of heart artery (Fig. 5) and histology of liver (Fig. 6) observed after treatment adds value to the above results by a clear depiction of some fields with reduced-fat droplets in the liver and reduced inflammatory cells in the artery of the heart.

## 4 Discussion

Ayurveda describes a vast array of herbs and herbal mixtures that have demonstrated to possess efficacy in research investigations. Kumar et al. (2012) observed the significant effect of Arjuna powder (*Terminalia arjuna*) and Arogyavardhini Vati (a polyherbal formulation) in dyslipidemic patients [22]. While Bhandari et al. (2002) study provides the potential effect of ethanolic extract of *Embelia ribes* on dyslipidemia in diabetic rats [23]. The drug GTG has been explored in the conditions like osteoarthritis [24] and inflammation [15]. Thus we explored the effect of the drug GTG on hyperlipidemia, hyperglycemia and inflammation in the present study.

The worldwide disease of obesity often occurs alongside several metabolic abnormalities, such as hypertension, hyperglycemia, and dyslipidemia. As a result of adipocyte hypertrophy, changes in adipocytokine profiles result in insulin resistance and inflammation, as well as changes in adipocytokine production [25].

Atherosclerosis is preceded by the deposition of VCAM-1, an adhesion molecule that exists in two forms in humans and mice. Human VCAM-1's, two intertwine variants end in a receptor with either a seven Ig-like domain protein or a six-domain VCAM-1 that lacks domain 4. Mouse VCAM-1 is a full-length seven-domain form with three domains linked to glycophosphatidylinositol for insertion within the plasma membrane [26]. By analyzing serum lipid profile, blood glucose, and serum VCAM-1 level, this study examined the effect of Gugguluthiktaka gritham on HFD-induced obese rats for the drugs hypolipidemic, hypoglycemic, and anti-inflammatory effects.

Animal models of CVD, including cardiac and atherothrombotic conditions, give vital understandings into the elaboration and pathophysiology of CVD, and they became essential tools to gauge new remedial strategies to prognosticate or to stop complications involved in the disease management [27]. Increased dietary fat fed to Wistar rats persuaded the cardiovascular, biochemical risk factors, and histological changes associated with a metabolic disease seen in humans. Precisely, high-fat diet ingestion enhanced bodyweight, stimulated hepatic and visceral and accumulation of fat [28].

Table 1 depicts the comparison of fasting blood glucose levels, serum lipid profile, and serum VCAM- 1 in the dyslipidemic group fed with a high-fat diet for a prolonged period of time with the control group. All the levels were significantly higher in the dyslipidemic group than those in the control group before treatment (p < 0.05) with mild elevation in the LDL and the observed results are taken as criteria to characterize dyslipidemia [[29], [30], [31]]. In dyslipidemia, change in lipid levels leads to insulin resistance and reduced efficiency of insulin leading to hyperglycemia and increased fat accumulation in the liver (as observed in our study findings). Due to IR, vasculature LPL shows suboptimal activity, which results in increased levels of TG in blood. To maintain TG homeostasis, liver VLDL production is increased. TG levels being high in the fasting state have also been shown to be independent predictors of CV disease [29,32,33].

The findings of HDL levels in Table 1, Table 2 might appear contradictory to the traditional definition of dyslipidemia since the dyslipidemic group shows higher HDL (Table 1) when compared to controls, while in Table 2, the test group shows higher HDL after treatment. In the last decade, a broad array of protective effects has been attributed to HDL, including reduced inflammatory and hemostatic effects. However, low HDL-c is not the only lipid disorder associated with dyslipidemia [29].

**Table 2:** Effect of the drug GTG on serum lipids, blood glucose, and serum VCAM-1 on dyslipidemia induced in Wistar rats.

Test parameter	BT	AT
TC (mg/dL)	156.33 ± 39.42	71.00 ± 11.12#
TG (mg/dL)	202 ± 81.61	92 ± 40.85∗
HDL (mg/dL)	94.16 ± 22.78	71.50 ± 51.39
LDL (mg/dL)	42.73 ± 27.59	42.80 ± 32.51
VLDL (mg/dL)	40.43 ± 16.32	18.43 ± 8.17∗
Blood glucose (mg/dL)	137 ± 20.73	53.66 ± 18.15^#^
VCAM 1 (ng/mL)	8.25 ± 1.02	4.14 ± 0.96^#^

BT = before treatment, AT = after treatment.

∗ = P < 0.05, # = P < 0.005 compared to before treatment in dyslipidemic group.

Values are expressed in mean ± SD.

New insights are emerging on HDL's role in atheroprotection. The atheroprotective effects of HDL cholesterol have been explored therapeutically, but results have been disappointing. According to a study, HDL cholesterol levels might not be a good predictor of MACE in CAD patients, i.e., serum levels of HDL cholesterol might not accurately reflect the true cholesterol efflux capacity of HDL. The researchers further found that the assessment of cholesterol efflux capacity provided the most reliable reflection of HDL function and cardiovascular risk and speculated that the disjunction between serum HDL concentration and its functional capacity might be caused by inflammation-induced remodeling of HDL subclass [34]. In another study, the cholesterol efflux capacity was reduced in ACS patients and remained low during the 3-month follow-up period when compared to stable CAD patients, independent of changes in HDL cholesterol levels or Apo lipoprotein A-I. These findings underscore the presence of atherogenic HDL dysfunction associated with acute coronary syndromes [35]. Interestingly, our findings depict the same picture and an increased level of HDL is observed in the dyslipidemic group.

Metabolic inflammation plays an important role in the development of dyslipidemia due to the overproduction of pro-inflammatory cytokines [36]. Hence increased levels of VCAM-1 are noted. The fatty degeneration, micro and macro vascular degeneration with intracellular fat droplets were noted in the histology of arteries of the heart and the liver when compared to a normal control group, confirming the development of dyslipidemia.

Table 2 represents the effect of the drug GTG after treatment on the dyslipidemic group. A significant reduction in total cholesterol (p < 0.002), Triglyceride (p < 0.020), and VLDL (p < 0.02) was observed aiding to the reduction in the fat accumulation with the highly significant hypoglycemic effect of fasting blood glucose (p < 0.001). As a result of our significant study findings, it has been found that GTG exerts a positive effect on fat and glucose metabolism when compared to inducing high-fat diets in experimental animals, which would otherwise compromise glucose tolerance and result in lipid changes [37]. A significance of p < 0.001 is noted for VCAM-1 depicting the reduction in inflammation which in turn would down-regulate ROS production thus reducing the risk of atherosclerotic plaque formation. Histology of heart artery and liver observed after treatment depicted reduced-fat droplets in the liver and reduced inflammatory cells in the artery of the heart providing a beneficial insight on the treatment of dyslipidemia by the drug GTG.

The effect of the drug GTG may be possibly due to reinstatement of the pancreatic cells or decreased intestinal absorption of glucose, enhanced uptake of glucose in cells, inhibition of progress of advanced glycated end-products, increase in liver glycogen and glucokinase activity, and antioxidant effects [38].

## 5 Conclusions

The present study concludes the therapeutic action of GTG in significantly lowering blood glucose levels contributing to its anti-hyperglycemic effect. The reduced total cholesterol, triglycerides, VLDL in blood, and the reduced-fat deposition in the histology of liver cells add value in managing dyslipidemia. A decrease in pro-inflammatory cytokine, VCAM-1, and mononuclear cells of inflammation in the histology of heart arteries possibly contributes to the effect of GTG in controlling the immune conditions. The study is limited by the unmet understanding of the molecular mechanism behind the drug's anti-hyperglycemic, anti-hyperlipidemic, and anti-inflammatory effects, which adds a possibility and scope for future studies in clinical settings.

## Author's contribution

**Samreen M Sheik:** Methodology, Formal analysis, Investigation, Writing – original draft **Pugazhandhi Bakthavatchalam:** Methodology, Investigation **Revathi P Shenoy:** Software, Validation, Writing – review and editing **Basavaraj S Hadapad:** Conceptualization **Deepak Nayak M:** Investigation **Monalisa Biswas:** Software, Validation **Varashree B S:** Resources, Writing – review and editing, Supervision, Project administration, Funding acquisition.

## Source of funding

Intramural Faculty Seed grant by the Directorate of Research, Manipal Academy of Higher Education (MAHE) (ID 00000031).

## Declaration of competing interest

None.
